# A New Element in the Blood

**Published:** 1898-03

**Authors:** 


### A New Element in the Blood.

H. F. Muller in the twenty-fifth volume of the *Centralbl. f. Path. und Brkteriol.*, p. 529, describes a hitherto unknown morphological element of the blood. It occurs in the plasma especially, near the red blood-cells. These elements are small, colorless, sometimes transparent, strongly refractive bodies, which show a marked motility in the fresh specimen. They are not stained by osmic acid, and thus are not fatty in their nature. They are not fibrin formations, since they are insoluble in acetic acid. The author has found them constantly in normal blood in different individuals and at all times. They vary, however, in number. In hungered and cachectic conditions they are diminished. The writer names them "haemokonien" or "blood-dust," and considers them as elementary granules, but gives no further characters.—*J. in American Medico-Surgical Bulletin.*

